# Implementation of an automated cluster alert system into the routine work of infection control and hospital epidemiology: experiences from a tertiary care university hospital

**DOI:** 10.1186/s12879-021-06771-8

**Published:** 2021-10-18

**Authors:** Seven Johannes Sam Aghdassi, Britta Kohlmorgen, Christin Schröder, Luis Alberto Peña Diaz, Norbert Thoma, Anna Maria Rohde, Brar Piening, Petra Gastmeier, Michael Behnke

**Affiliations:** 1 Institute of Hygiene and Environmental Medicine, Charité-Universitätsmedizin Berlin, Universität zu Berlin, Hindenburgdamm 27, 12203 Berlin, Germany; 2 National Reference Centre for Surveillance of Nosocomial Infections, Hindenburgdamm 27, 12203 Berlin, Germany

**Keywords:** Automation, Digitalization, Cluster alert system, Outbreak, Infection control, Hospital epidemiology

## Abstract

**Background:**

Early detection of clusters of pathogens is crucial for infection prevention and control (IPC) in hospitals. Conventional manual cluster detection is usually restricted to certain areas of the hospital and multidrug resistant organisms. Automation can increase the comprehensiveness of cluster surveillance without depleting human resources. We aimed to describe the application of an automated cluster alert system (CLAR) in the routine IPC work in a hospital. Additionally, we aimed to provide information on the clusters detected and their properties.

**Methods:**

CLAR was continuously utilized during the year 2019 at Charité university hospital. CLAR analyzed microbiological and patient-related data to calculate a pathogen-baseline for every ward. Daily, this baseline was compared to data of the previous 14 days. If the baseline was exceeded, a cluster alert was generated and sent to the IPC team. From July 2019 onwards, alerts were systematically categorized as relevant or non-relevant at the discretion of the IPC physician in charge.

**Results:**

In one year, CLAR detected 1,714 clusters. The median number of isolates per cluster was two. The most common cluster pathogens were *Enterococcus faecium* (n = 326, 19 %), *Escherichia coli* (n = 274, 16 %) and *Enterococcus faecalis* (n = 250, 15 %). The majority of clusters (n = 1,360, 79 %) comprised of susceptible organisms. For 906 alerts relevance assessment was performed, with 317 (35 %) alerts being classified as relevant.

**Conclusions:**

CLAR demonstrated the capability of detecting small clusters and clusters of susceptible organisms. Future improvements must aim to reduce the number of non-relevant alerts without impeding detection of relevant clusters. Digital solutions to IPC represent a considerable potential for improved patient care. Systems such as CLAR could be adapted to other hospitals and healthcare settings, and thereby serve as a means to fulfill these potentials.

## Background

Infection prevention and control (IPC) is a cornerstone of quality management and ensuring the safety of patients in hospitals [1, 2]. Principal objectives of IPC are preventing healthcare-associated infections and reducing pathogen transmission [3–5]. To achieve these objectives, timely detection of and effective mitigation against healthcare-associated outbreaks are imperative. Healthcare-associated outbreaks frequently are the result of an uncontrolled spread of pathogens within a healthcare facility [6–8]. Consequently, early detection of pathogen spread (i.e. clusters) is a prerequisite for effective outbreak management and containment.

Conventionally, cluster detection in hospitals is a laborious manual process that requires substantial investment of time and human resources [9]. In most German hospitals, cluster detection falls into the responsibility of IPC staff that often has limited resources available. As a result, cluster monitoring is usually confined to certain “risk areas” within a hospital (e.g. intensive care units, transplant units, neonatology) and to pathogens with specific attributes (e.g. multidrug resistant organisms (MDROs)). This restrictive approach results in a significant “blind spot” and possible negligence of susceptible organisms and non-critical areas of a hospital that can be equally affected by outbreaks. Another deficit of the current manual approach is the frequently arbitrary definition of what constitutes a cluster. In many cases, subjective criteria are applied, such as a certain number of isolates in a defined period, without considering the context and the endemic level of a pathogen.

Automated cluster alert systems offer an opportunity to improve current practices in cluster detection [10–12]. They can serve as a way to establish hospital-wide cluster surveillance of a broad range of pathogens that can be both MDROs and susceptible organisms. Contrary to current practices, automated cluster alert systems present possibilities to detect clusters in a reproducible and objective manner [13, 14]. Despite increasing the amount of data processed, alert systems could reduce the workload for IPC staff by automating certain steps in the cluster detection workflow and thereby save time and human resources.

This study presents data from and experiences with an automated cluster alert system that was incorporated into the daily routine IPC work of a tertiary care hospital in Germany. The objectives of this study were to describe the use of the automated cluster alert system by the local IPC team in a clinical routine setting, as well as to provide an overview about the clusters detected and their properties from a one-year period of continuous use.

## Methods

### Setting

Charité university hospital is a tertiary care hospital with over 3,000 patient beds that is located at three separate sites in Berlin, Germany. The IPC team constitutes of IPC nurses and physicians that are jointly responsible for the detection of nosocomial clusters and coordinating mitigation efforts when relevant clusters are detected. Owing to healthcare-associated outbreaks in the past, a decision was made to develop an automated cluster alert system to improve cluster detection and pathogen surveillance. The system was named CLAR (cluster alarm system), introduced into the IPC work of the hospital in November 2017, and following various adjustments, successively incorporated into the routine work in 2018. In the year 2019, the system was continuously in use and only underwent marginal changes. We therefore decided to focus our analyses on the data generated during that period.

Hospitals in Germany are required by the German Protection Against Infection Act to collect surveillance data on healthcare-associated infections and certain pathogens [15]. Since the data utilized by CLAR were collected in alignment with this regulation, ethical approval and informed consent were not required.

### Overview of the functions of the automated cluster alert system

CLAR reviewed and analyzed routinely collected microbiological and patient-related data (e.g. patient movement) that converged and were stored in a data warehouse. CLAR utilized data of the previous two years from the data warehouse to calculate a baseline for every included pathogen at every hospital ward. Daily, this baseline was compared to data from a period of the previous 14 days. By employing six different algorithms, CLAR evaluated whether the number of detected isolates at a ward during the previous 14 days exceeded the two-year baseline. The algorithms utilized for this purpose were normal distribution prediction intervals (PI-NV), Poisson distribution (PI-POI) and score prediction intervals (PI-SCORE) for interval prediction, early aberration reporting system (EARS) and negative binominal CUSUMs (NBC) for statistical process control, and Farrington algorithm for statistical modelling. The specifics of the applied algorithms as well as their utilization within the data warehouse have been described in a previous publication [11]. Where available, resistance profile data of pathogens were included and only isolates, for which the intervals of the minimum inhibitory concentration for tested antibiotics overlapped, were considered. Where resistance information was not available, isolates of any phenotype were considered. If the baseline was exceeded, CLAR generated an alert email that was sent to the responsible IPC physician for review. From 10 to 2019 onwards, all alerts were labelled as either relevant or non-relevant by the IPC physician in charge. Relevance in this context denoted that the alert triggered measures (e.g. further investigation, IPC training, genotyping, outbreak management) at the respective ward.

### Eligibility of pathogens and isolates

The following pathogens or groups of pathogens were considered by CLAR, both for generating the two-year baseline and for evaluating the previous 14 days: *Acinetobacter baumannii*, *Clostridioides difficile*, *Citrobacter* spp., *Escherichia coli*, *Enterococcus faecalis*, *Enterococcus faecium*, *Enterobacter* spp., *Klebsiella* spp., *Pseudomonas aeruginosa*, *Staphylococcus aureus*, *Salmonella* spp. and *Serratia* spp. Additionally, all pathogens cultivated from blood cultures were included. To place a focus on nosocomial clusters, only isolates that were sampled at least two days after admission to the ward were included. A separate rule for blood cultures also considered isolates sampled prior to the second day after admission. Copy strains (i.e. the same pathogen was detected in the same patient multiple times) were excluded for a duration of 90 days.

### Data analysis

Alerts generated by CLAR between 1 and 2019 and 31 December 2019 were included in the analyses. Only alerts pertaining to in-patient areas of the hospital and alerts pertaining to a single ward were included. For every alert, the number of detected isolates, type of pathogen and resistance information, sampling material, and ward at which the alert occurred were recorded. For data presentation in this article, wards were separated into adult intensive care units (ICUs), adult non-ICUs and neonatal and pediatric (NEOPED) units. Sampling materials were distinguished into clinical (e.g. blood culture, wound swab) and screening (e.g. rectal swab) isolates.

When presenting data concerning the clinical relevance of alerts as assessed by the IPC physician, only alerts for which this information was recorded (10 July 2019 – 31 December 2019) were considered. Alerts during this period, for which no assessment of relevance was documented (missing data), were excluded from the analysis focusing on alert relevance. Differences regarding the relevance of alerts were tested by univariable analysis using a two-sided Chi-squared test. Analyses were conducted with OpenEpi [16]. A p-value of less than 0.05 was considered statistically significant.

## Results

### Overview

A total of 1,009,051 patient days were generated at Charité university hospital in the year 2019, 822,021 of which pertained to adult non-ICUs, 85,269 to adult ICUs, and 101,761 to NEOPED units. During the observed period, CLAR detected 1714 clusters for which an alert notification was generated, which is equivalent to 1.7 alerts per 1,000 patient days. Alert occurrence per 1,000 patient days was 1.2 for adult non-ICUs, 7.4 for adult ICUs, and 1.2 for NEOPED units.

Almost all clusters that were detected (n = 1603, 94 %) contained at least one clinical isolate, while 6 % (n = 111) clusters solely included screening isolates. Around 21 % (n = 354) of all detected clusters included at least one multidrug resistant isolate, while 79 % (n = 1360) of clusters contained only susceptible pathogens. The majority of detected clusters comprised of three or less isolates (n = 1456, 85 %). The average and median number of isolates per alert was 2.7 and 2 respectively. When stratifying by ward type, similar distributions concerning the number of isolates per alert were observed. Table 1 summarizes the frequency of detected clusters stratified by cluster size and type of ward.

**Table 1:** Clusters detected by the automated cluster alert system in the year 2019

Cluster size	All wards	Adult non-ICUs	Adult ICUs	Neonatal/pediatric wards
	Number (%) or Value	Number (%) or Value	Number (%) or Value	Number (%) or Value
All	1714 (100)	966 (100)	628 (100)	120 (70)
2	1026 (59.9)	592 (61.3)	364 (58.0)	70 (58.3)
3	430 (25.1)	247 (25.6)	160 (25.5)	23 (19.2)
4	150 (8.8)	81 (8.4)	57 (9.1)	12 (10)
5	52 (3.0)	22 (2.3)	21 (3.3)	9 (7.5)
6	33 (1.9)	12 (1.2)	16 (2.5)	5 (4.2)
7	9 (0.5)	2 (0.2)	7 (1.1)	0 (0)
8	10 (0.6)	6 (0.6)	3 (0.5)	1 (0.8)
>8	4 (0.2)	4 (0.4)	0 (0)	0 (0)
First quartile	2	2	2	2
Median	2	2	2	2
Third quartile	3	3	3	3

Stratification by cluster size (number of isolates) and ward type. *ICU* intensive care unit

### Microorganisms

**Fig. 1:**
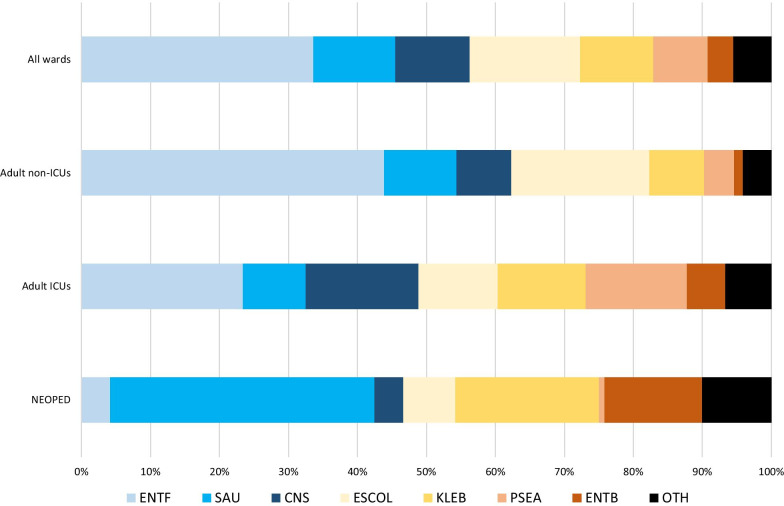
Clusters detected by the automated cluster alert system in the year 2019. Stratification by pathogen and ward type. *CNS* coagulase-negative staphylococci, *ENTB* *Enterobacter* spp., *ENTF* *Enterococcus faecium* and *Enterococcus faecalis*, *ESCOL* *Escherichia coli*, *ICU* intensive care unit, *KLEB* *Klebsiella* spp., *NEOPED* neonatology and pediatrics, *OTH* other pathogens, *PSEA* *Pseudomonas aeruginosa*, *SAU* *Staphylococcus aureus*

The highest number of alerts generated were due to clusters of *E. faecium* (n = 326, 19 %), *E. coli* (n = 274, 16 %) and *E. faecalis* (n = 250, 15 %). When differentiating by ward type, differences were observed concerning the pathogens most frequently found in clusters. Adult non-ICUs (total number of alerts: 966) yielded a similar pathogen distribution, with clusters of *E. faecium* (n = 212, 22 %), *E. faecalis* (n = 212, 22 %) and *E. coli* (n = 193, 20 %) being the most commonly detected. While *E. faecium* (n = 112, 18 %) was also the most commonly detected cluster pathogen in adult ICUs (total number of alerts: 628), clusters of coagulase-negative staphylococci and Gram-negative pathogens were detected more frequently than in non-ICUs. Clusters detected in NEOPED units (total number of alerts: 120) were revealed to have a different pathogen distribution than clusters in adult wards. *E. faecium* and *E. faecalis* jointly only accounted for 4 % (n = 5) of the clusters found, while *S. aureus* (n = 46, 38 %), *Klebsiella* spp. (n = 25, 21 %) and *Enterobacter* spp. (n = 17, 14 %) were the most commonly detected cluster pathogens. Table 2; Fig. 1 illustrate the pathogen distribution of clusters detected by CLAR in relation to the ward type.

**Table 2:** Clusters detected by the automated cluster alert system in the year 2019

Pathogen (-group)	All wards	Adult non-ICUs	Adult ICUs	Neonatal/pediatric wards
	Number (%)	Number (%)	Number (%)	Number (%)
All	1714 (100)	966 (100)	628 (100)	120 (70)
*Enterococcus faecium*	326 (19.0)	212 (21.9)	112 (17.8)	2 (1.7)
*Enterococcus faecalis*	250 (14.6)	212 (21.9)	35 (5.6)	3 (2.5)
*Staphylococcus aureus*	204 (11.9)	101 (10.5)	57 (9.1)	46 (38.3)
CNS	185 (10.8)	77 (8.0)	103 (16.4)	5 (4.2)
*Escherichia coli*	274 (16.0)	193 (20.0)	72 (11.5)	9 (7.5)
*Klebsiella* spp.	182 (10.6)	77 (8.0)	80 (12.7)	25 (20.8)
*Pseudomonas aeruginosa*	135 (7.9)	42 (4.3)	92 (14.6)	1 (0.8)
*Enterobacter *spp.	64 (3.7)	12 (1.2)	35 (5.6)	17 (14.2)
*Serratia* spp.	23 (1.3)	2 (0.2)	14 (2.2)	7 (5.8)
*Clostridioides difficile*	43 (2.5)	31 (3.2)	11 (1.8)	1 (0.8)
*Candida* spp.	13 (0.8)	3 (0.3)	10 (1.6)	0 (0)
*Acinetobacter baumannii*	6 (0.4)	0 (0)	2 (0.3)	4 (3.3)
*Citrobacter* spp.	4 (0.2)	2 (0.2)	2 (0.3)	0 (0)
*Streptococcus* spp.	2 (0.1)	2 (0.2)	0 (0)	0 (0)
*Proteus* spp.	1 (0.1)	0 (0)	1 (0.2)	0 (0)
*Micrococcus* spp.	1 (0.1)	0 (0)	1 (0.2)	0 (0)
*Clostridium perfringens*	1 (0.1)	0 (0)	1 (0.2)	0 (0)

Stratification by pathogen and ward type. *CNS* coagulase-negative staphylococci, *ICU* intensive care unit

### Relevance of alerts

From 10 to 2019 onwards, the relevance of alerts was systematically documented by IPC physicians. Between 10 and 2019 and 31 December 2019, CLAR generated 924 alerts (54 % of all alerts in 2019). For 906 (98 %) of these 924 alerts documentation of relevance assessment was performed. Overall, 589 (65 %) of alerts assessed were deemed non-relevant. Conversely, 317 (35 %) alerts were classified as being clinically relevant and triggered further cluster-related investigations or measures.

When comparing the characteristics of relevant and non-relevant alerts, various differences were noted. Relevant alerts tended to contain a greater number of isolates then non-relevant alerts. The percentage of alerts with more than three isolates was significantly higher in the group of relevant alerts (24 %) than non-relevant alerts (18 %) (p = 0.02). Relevant alerts pertained significantly more often to ICUs (51 %) than alerts deemed non-relevant (31 %) (p < 0.01). For non-ICUs, an inverse relation was seen. Regarding the correlation between pathogen and alert relevance, diverse results were observed. While the percentage of *Klebsiella* spp. and *Enterobacter* spp. clusters detected by CLAR was significantly higher in the group of relevant alerts in comparison to non-relevant alerts, it was significantly lower for clusters of *Enterococcus* spp., coagulase-negative staphylococci and *E. coli*. No significant differences were observed between relevant and non-relevant alerts with regards to whether an alert contained at least one clinical isolate or at least one MDRO. A detailed illustration of the comparison between relevant and non-relevant alerts can be found in Table 3.

**Table 3:** Clusters detected by the automated cluster alert system in the year 2019 with assessment of relevance by infection control physicians (n = 906)

Parameter	Property	Relevant alerts	Non-relevant alerts	Significance
		Number (%)	Number (%)	p-value (two-tailed)
Cluster size (number of isolates)	All	317 (100)	589 (100)	n.a.
	2	154 (48.6)	312 (53.0)	0.23
	3	86 (27.1)	173 (29.4)	0.53
	>3	77 (24.3)	104 (17.7)	0.02
Ward type	All	317 (100)	589 (100)	n.a.
	Adult non-ICU	131 (41.3)	377 (64.0)	<0.01
	Adult ICU	162 (51.1)	184 (31.2)	<0.01
	NEOPED	24 (7.6)	28 (4.8)	0.11
Pathogen(-group)	All	317 (100)	589 (100)	n.a.
	*Enterococcus* spp.	86 (27.1)	206 (35.0)	0.02
	*Staphylococcus aureus*	34 (10.7)	59 (10.0)	0.83
	CNS	25 (7.9)	123 (20.9)	<0.01
	*Escherichia coli*	29 (9.1)	100 (17.0)	<0.01
	*Klebsiella* spp.	70 (22.1)	43 (7.3)	<0.01
	*Pseudomonas aeruginosa*	23 (7.3)	33 (5.6)	0.40
	*Enterobacter* spp.	15 (4.7)	11 (1.9)	0.02
	Other	35 (11.0)	14 (2.4)	<0.01
Cluster includes at least one clinical isolate	All	317 (100)	589 (100)	n.a.
	Yes	290 (91.5)	557 (94.6)	0.10
	No	27 (8.5)	32 (5.4)	0.10
Cluster includes at least one multidrug resistant isolate	All	317 (100)	589 (100)	n.a.
	Yes	75 (23.7)	137 (23.3)	0.96
	No	242 (76.3)	452 (76.7)	0.96

P-values were calculated using two-sided Chi-squared test. Sampling materials were distinguished into clinical (e.g. blood culture, wound swab) and screening (e.g. rectal swab) isolates. Univariable analysis of characteristics. *CNS* coagulase-negative staphylococci, *ICU* intensive care unit, *n.a.* not applicable, *NEOPED* neonatology and pediatrics

## Discussion

The automated cluster alert system implemented at Charité university hospital was continuously used in the year 2019 and steadily generated cluster alerts during this period. Unlike most other studies focusing on automated cluster detection [17–19], the data presented in this article, stem from real-life routine utilization in a large hospital and were collected prospectively. Data from routinely used automated cluster alert systems are scarce, thus, it was our intention to delineate the experiences with our system in order to reduce this knowledge gap.

By taking into account baseline information on pathogen occurrence from the previous two years, which was then compared to the number of isolates of a specific pathogen during the previous 14 days, CLAR was based upon objective and reproducible criteria. Since cluster detection in our hospital before the implementation of CLAR was not systematized in an equal manner, it is not possible to specify the exact number of clusters that were detected in the years prior to CLAR utilization. However, based on our own experiences, we can confidently state that this number was considerably lower than the number of clusters detected by CLAR in the year 2019. The high number of clusters detected by CLAR indicates a high degree of sensitivity. The majority of clusters detected by CLAR were caused by susceptible organisms. Manual cluster detection, on the other hand, is conventionally focused on MDROs [20, 21], which could be a reason that the number of clusters detected by CLAR in 2019 was higher than the number detected by manual detection in previous years. Our findings illustrate, however, that omitting susceptible organisms from cluster surveillance can result in missing many potentially relevant clusters. From July 2019 onwards, alerts were evaluated by IPC physicians concerning their clinical relevance. The proportion of alerts with at least one MDRO among all relevant alerts was almost identical to the proportion of alerts with MDROs among all non-relevant alerts (24 % vs. 23 %). This demonstrates that the higher number of alerts with susceptible organisms was not just “debris data” that yielded no clinical value, but substantially contributed to IPC practice in our hospital.

Another aspect that confirms the high sensitivity of CLAR is the fact that the majority of clusters detected consisted of a rather low number of isolates (i.e. two or three). Although the proportion of larger cluster (i.e. over three isolates) was significantly higher among relevant than non-relevant alerts, a considerable number of detected clusters with only two or three isolates were deemed relevant by IPC physicians, and in some cases measures were taken to mitigate the spread of the pathogen. Given that even large outbreaks initially begin with a small number of cases, it is conceivable that some of these small clusters that were detected early might have resulted in larger outbreaks, had they not been brought to the attention of the IPC team by CLAR. Nevertheless, it has to be acknowledged that around two thirds of all alerts with relevance assessment were deemed non-relevant. Therefore, alert specificity is an important point for improvement of CLAR. This aspect is particularly important since the workload and time invested into evaluating non-relevant alerts might distract from adequately focusing on relevant alerts.

Since CLAR employed pathogen specific rules, we were able to collect data on the types of pathogens and pathogen-groups that accounted for the highest number of clusters. *E. faecium* and *E. faecalis* as well as *E. coli* clusters were the most frequently identified, reinforcing evidence that these pathogens are commonly causing nosocomial outbreaks [22, 23]. The differences that were revealed by distinguishing between different types of wards, illustrate that the likelihood of pathogens to cluster is not the same hospital-wide, but varies by patient population and type of care. The observation that Gram-negative bacteria, such as *Klebsiella* spp., *Enterobacter* spp. and *Serratia* spp. can be particularly problematic in pediatric and neonatal settings is in alignment with data from previous publications [24, 25].

Conventional cluster detection places a focus on patients in ICUs and other critical areas of a hospital [26]. Our findings revealed that this focus is to a certain extent justified, as the number of alerts per 1,000 patient days was much higher in adult ICUs compared to other ward types and the percentage of ICU alerts that were deemed relevant was significantly higher than for non-ICU alerts. However, a considerable proportion of non-ICU alerts were classified as relevant, highlighting that non-ICUs should not be neglected when conducting cluster surveillance. The fact that during a period of roughly six months over 300 alerts were deemed relevant, represents a remarkable finding. When extrapolated to a one-year period at a 3,000-bed hospital, we can estimate that around 20 relevant alerts per 100 patient beds might occur annually.

Statistical analysis yielded diverse results considering the association of alert relevance and type of pathogen. Since the data available were only from a one-year period and in some cases only included a low number of isolates, these findings should be interpreted with caution. However, the high percentage of clusters of *Klebsiella* spp. and *Enterobacter* spp. among relevant alerts reinforce experiences that Gram-negative bacteria can be particularly challenging in the practice of IPC in an acute care hospital. Healthcare-associated infections caused by Gram-negative bacteria have been described as a concern to patient safety [27]. Automated cluster alert systems can serve as a tool for early detection of such infections.

The comprehensive approach of CLAR that includes susceptible organisms and monitors cluster occurrence in all wards of a hospital may show its true merit in situations when human healthcare resources are scarce or need to be re-allocated. The COVID-19 pandemic has demonstrated how quickly human resources can become scarce and how demands on employees can change [28]. To have a robust, autonomous system in place that ensures a continuous and steady quality of cluster monitoring could be a great advantage in such situations.

Digital solutions for streamlining workflows in the practice of medicine in general, and IPC more specifically, have gained recognition in recent years [29]. Regarding automated cluster detection however, potentials of digitalization are not yet fulfilled. Successful applications of automated cluster alert systems, such as CLAR at all three sites of Charité university hospital, may serve as an incentive for other hospitals to establish similar systems. Furthermore, data generated and evaluated by automated cluster alert systems such as CLAR, may facilitate the exchange of data between institutions as well as the transfer of information from hospitals to public health organizations. CLAR was implemented at a hospital that had established routines regarding the practice of IPC at the time of the implementation. We consider the pre-existence of a dedicated IPC department and IPC-related processes to be a prerequisite for the successful implementation of any automated cluster alert system.

Various limitations have to be acknowledged when interpreting the data. CLAR identified clusters by comparing a 14-day period to a baseline that was established by analyzing data of the previous 2 years. Outbreaks that have occurred during these two years might have artificially increased the baseline in some cases. To counteract this potential confounder, six different algorithms were employed in order to compensate for possible outbreak-related baseline distortions. Although copy strains were eliminated by CLAR, in situations where clusters gradually increased in size (e.g. n = 2 on day one; n = 3 on day four), multiple alerts might have been generated that contained identical isolates. Therefore, the number of clusters detected by CLAR cannot be uncritically equated with clustering events at a ward. Moreover, it is important to recognize that not all clusters detected by CLAR likely represented nosocomial transmissions, but in some cases rather might have been attributable to random fluctuations in pathogen occurrence at a ward. In order to verify whether isolates in clusters detected by CLAR were genetically identical and thus likely attributable to transmission events, genome sequencing would be necessary. The relevance assessment of alerts was done at the discretion of the IPC physician in charge for the ward that the alert pertained to. The IPC physicians at Charité university hospital during that time were a diverse group of professionals at different stages of their training. Despite regular team meetings where questions about CLAR were discussed and alerts were worked on jointly to ensure a high degree of consistency, individual differences in how alert data were evaluated could represent a confounder.

## Conclusions

The automation of cluster detection offers great potentials for the practice of infection control. The automated cluster alert system in use in our hospital represents a viable alternative to conventional manual cluster detection and was able to identify a high number of alerts that were deemed relevant by IPC physicians. Automated cluster alert systems can help detecting healthcare-associated clusters early and thereby serve as an effective tool to prevent the uncontrolled spread of pathogens in a hospital. Particularly clusters of susceptible pathogens that might otherwise not be noticed early, were detected reliably by the automated cluster alert system. We consider the ability of our alert system to detect even small clusters that deviate from the baseline for a pathogen at a ward to be a great benefit for patient safety. However, potentials for improvement remain regarding the specificity of alerts. A target for future developments must therefore be to reduce the number of non-relevant alerts without impeding the detection of clinically relevant clusters. Further research is required to reconcile these two objectives.

## Data Availability

The datasets used and/or analyzed during the current study are available from the corresponding author upon reasonable request.

## Abbreviations

CLAR: Cluster alarm system
ICU: Intensive care unit
IPC: Infection prevention and control
MDRO: Multidrug resistant organism
NEOPED: Neonatal and pediatric
